# Very Long Chain Marine *n*-3 Polyunsaturated Fatty Acids in Atherothrombotic Heart Disease. A Brief Review, with a Focus on Metabolic Effects

**DOI:** 10.3390/nu12103014

**Published:** 2020-09-30

**Authors:** Harald Arnesen, Peder L. Myhre, Ingebjørg Seljeflot

**Affiliations:** 1 Center for Clinical Heart Research, Department of Cardiology, Oslo University Hospital Ullevål, Pb 4956 Nydalen, 0424 Oslo, Norway; harald.arnesen@ous-hf.no; 2 Faculty of Medicine, University of Oslo, 0424 Oslo, Norway; p.l.myhre@medisin.uio.no; 3 Division of Medicine, Department of Cardiology, Akershus University Hospital, 1478 Lørenskog, Norway

**Keywords:** omega-3 fatty acids, metabolism, inflammation, atherothrombosis, clinical trials

## Abstract

The global burden of atherothrombotic heart disease should be considered as a life-style disorder where differences in dietary habits and related risk factors like limited physical activity and adiposity together play important roles. Related metabolic changes have been scientifically elucidated in recent decades, and the role of the very-long-chain marine fatty acids eicosapentaenoic acid (EPA) and docosahexaenoic acid (DHA) have been much focused on, especially their possible effects on processes like inflammation and thrombosis. In the present brief review of related metabolic mechanisms, the effects of these fatty acids in a clinical setting have been referred to, including some of the authors’ work on this topic. The main focus is the divergent results in the field and the important differences between the study population, the type of supplements and fresh marine sources, the proportion of EPA versus DHA dosages, and the duration of supplementation in clinical trials. We conclude that daily intake of at least 1 g of EPA + DHA may improve a dysmetabolic state in the population. The potential to reduce the risk and progression of atherothrombotic heart disease is still a matter of debate.

## 1. Background

Atherothrombotic heart disease is a dominating cause of morbidity and mortality worldwide [1,2]. Atherothrombotic heart disease comprises the continuous process of atherosclerosis, leading to narrowing of the coronary arteries and possibly the clinical manifestation angina pectoris, and the acute thrombotic occlusion of the artery as a basis for the life-threatening myocardial infarction [3,4]. Lifestyle factors like dietary habits have been highlighted as risk factors for atherothrombosis [5,6,7]. Preventive approaches like achievable dietary changes are obviously of major importance both for health and economic reasons. The importance of increased intake of very-long-chain *n*-3 polyunsaturated fatty acids (VLCM *n*-3 PUFAs), especially from marine sources, has been discussed over the last 50 years, back to the observations on Greenland Eskimos by the Danish scientists Dyerberg and Bang [8,9]. They found that the occurrence of coronary heart disease was very low among the Eskimos compared to the regular Danish population, and they attributed this observation to their special diet, which was composed mainly of seal and whale and was extremely rich in marine *n*-3 FAs. Later, a series of mechanisms for the potentially beneficial effects of such VLCM *n*-3 PUFA were published [10,11,12,13,14,15].

**Figure 1:**
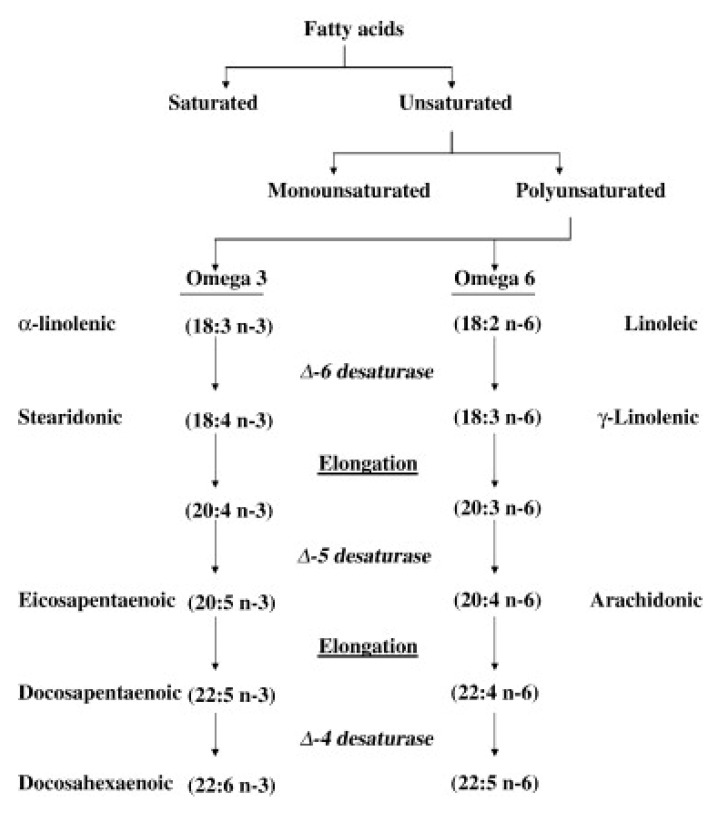
Metabolic pathway of omega-6 and omega-3 fatty acid synthesis. Copied from Siddiqui RA, Harvey KA, Zaloga GP. *J. Nutr. Biochem.* **2008**, *19*, 417–437 [10], with permission.

Relevant biologically active families of polyunsaturated FAs are the *n*-6 and *n*-3 FAs that contribute to several important metabolic pathways. However, as mammals lack enzymes to insert double bonds in the *n*-6 and *n*-3 position, linoleic acid (LA) (18:2 *n*-6) and alfa-linolenic acid (ALA) (18:3 *n*-3) are essential nutrients. In addition, the elongation and desaturation of these FAs to the metabolically important VLCM PUFAs are limited, and the *n*-3 and *n*-6 series are competing for the same enzymes, the elongases, and the desaturases (Figure 1). The metabolic key FAs in the two series are arachidonic acid (AA) (20:4 *n*-6) in the *n*-6 series and eicosapentaenoic acid (EPA) (20:5 *n*-3) and docosahexaenoic acid (DHA) (22:6 *n*-3) in the *n*-3 series [12,13,15].

In regular Western diet today, the content of LA (from green plants and red meat) is much higher than that of ALA. Thus, the competition for elongases and desaturases favors the synthesis of the *n*-6 series, i.e., mainly AA, which is metabolized into mainly pro-inflammatory and platelet activating derivatives. Therefore, the synthesis of the very-long-chain EPA and DHA are minimal, especially when the diet is relatively low in ALA [16,17]. Derivatives from EPA/DHA seem to be rather neutral with respect to inflammation and platelet activation, but contribute importantly to the resolution of inflammation by being so-called resolvins [13,18].

In recent decades, in addition to epidemiologic and registry studies, a series of observational and randomized clinical trials on the possible clinical effect of intake (dietary or as supplements) of VLCM *n*-3 PUFA have been conducted [13,14,15]. Additionally, the effects on in vivo VLCM *n*-3 PUFA in the circulation, adipose tissue, and erythrocyte membrane have been extensively studied [19,20,21]. The results are divergent, mainly depending on (i) the population studied, i.e., baseline health status and risk factors, primary versus secondary prevention, and background dietary intake of *n*-3 and *n*-6 FAs; (ii) the type of supplementation (fatty fish, cod liver oil, seal oil, or capsules); (iii) duration of supplementation; (iv) dosage of supplementation; (v) proportion of EPA and DHA; and (vi) the quality of capsule content.

As in all therapeutic trials, compliance with the investigational principle is relevant to assess the validity of the results. Dietary questionnaires are often used to characterize the diet, but are generally inadequate for scientific purposes, although validated scoring systems are available. In studies using supplementary capsules, “pill count” is often used as a measure of compliance. However, this approach has important scientific limitations relating to logistics and patient reliability. In addition, supplementary capsules are prone to peroxidation of the unsaturated FAs, although often add anti-oxidants. Thus, in vivo measurements of the relevant FAs in the circulation, erythrocyte membrane, and adipose tissue are considered to be the scientifically most reliable methods. This strategy also allows for the estimation of relevant indexes (EPA + DHA/total FAs % and *n*-6/*n*-3 ratios) [21,22,23].

It should be emphasized that the unsaturated FAs are prone to the peroxidation and saturation of their double bonds, potentially reducing their beneficial effects in atherosclerotic cardiovascular disease states [24,25]. This is often the case with commercial supplements of VLCM *n*-3 PUFAs if not enriched with antioxidants like vitamin E. Fresh marine fish products do not experience the same risk of peroxidation.

## 2. VLCM *n*-3 FA, Metabolic Disorders and Atherothrombotic Heart Disease

The metabolic syndrome is composed of the most important metabolic disorders related to atherothrombotic heart disease. It describes a cluster of risk factors for cardiovascular disease states (CVD) and diabetes type 2, including abdominal obesity (waist circumference ≥ 102 (men)/88 cm (women)), elevated fasting blood glucose (≥5.6 mmol/L), hypertension (blood pressure ≥ 130/85 or on treatment), elevated triglycerides (≥1.7 mmol/L), and low levels of HDL cholesterol (<1.0 (men)/1.3 (women) mmol/L), according to the scientific statement from the American Heart Association and National Heart, Lung, and Blood Institute [26].

Among these risk factors, triglycerides and hypertension have been given most attention as relevant targets for potential effects of the VLCM *n*-3 PUFA. Regarding triglycerides, fasting samples are most frequently used, although non-fasting values have lately been used in epidemiological studies [27]. Triglycerides have been repeatedly shown to be predictive of future CVD, and their importance in risk prediction has increased in parallel with the increased prevalence of the metabolic syndrome in the Western world. Already in 1992, this effect was demonstrated in two large population studies from Germany and Finland [28,29], and later was documented in several populations with long-term follow-up, like the PROCAM study [30]; the Oslo Study [31]; and, more recently, the Copenhagen Study [27].

Furthermore, the triglyceride-lowering effect of the VLCM *n*-3 PUFAs has been observed in numerous clinical trials and is considered a key pathway in preventing CVD. The mechanism by which these VLCM *n*-3 PUFAs reduce triglycerides is probably by reduced hepatic synthesis and secretion [32], and reduction of triglycerides has often been used as a measure of compliance in clinical trials. The dose-relationship is well known and is evident in the various clinical trials using a range of VLCM *n*-3 PUFA doses. In the GISSI-Prevenzione trial [33] using a low dose (0.85 g/day), the reduction in triglycerides was only 3.4% over 5.5 years, whereas in the CART study [34] with a very high dose of 5.1 g/day, the reduction over 1 year was 27%. In the SHOT [35] and DOIT [36] studies, relatively high doses of 3.4 g/day and 2.4 g/day, respectively, were used, resulting in 19 and 16% triglyceride reduction, respectively. In the recent REDUCE-IT study [37], the reduction with 4 g/day of icosapent ethyl (an ethyl-EPA that is metabolized to EPA after ingestion) over 4.9 years was 18%. In the latter study, the reduction of cardiovascular risk could not be explained solely by the reduction in triglycerides, suggesting other cardio-protective mechanisms from high-dose EPA [38]. In the 2019 European Society of Cardiology/European Atherosclerosis Society guidelines VLCM *n*-3 PUFA has a IIa recommendation for lowering triglycerides [39]. High doses of the VLCM *n*-3 PUFA are recommended for very high levels of triglycerides, mainly to avoid pancreatitis [40]. Whether even higher doses, e.g., 8–12 g/day would have even better longstanding effects is unclear, but at these doses bowel dysfunction may be a challenge.

In lipidology, there is a well-known inverse relationship between triglycerides and HDL-cholesterol, although this may differ between subtypes of HDL-cholesterol. Thus, with high levels of triglycerides low levels of HDL-cholesterol are regularly found. In studies on supplementation with VLCM *n*-3 PUFAs, the reduction in triglycerides is regularly accompanied by a moderate increase in HDL-cholesterol. A more specific effect on the synthesis or secretion of the latter has, however, not been clearly elucidated.

With respect to hypertension, the VLCM *n*-3 PUFAs have been shown to reduce both the systolic and diastolic blood pressure. Already in 1990, Bønaa et al. [41] could demonstrate in a randomized, control trial in 156 hypertensive men and women, a significant, although moderate, reduction of both SBP and DBP after six weeks supplementation with 5.1 g/day, and the effect was related to the measured increase in plasma *n*-3 FAs. Similar effects have later been demonstrated in larger meta-analyses [20,42]. 

**Figure 2:**
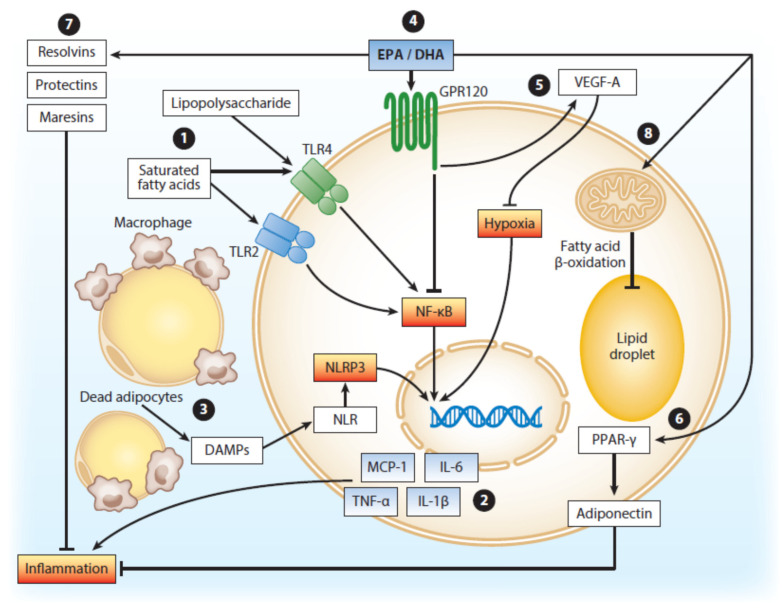
Modulation of white adipose tissue function by omega-3 polyunsaturated fatty acids (ω-3 PUFAs). Copied from Kalupahana NS et al., with permission. *Annu. Rev. Nutr.* **2020** Jun 16. doi:10.1146/annurev-nutr-122319-034142 [44].

In the metabolic syndrome, hyperglycemia and insulin resistance are linked to abdominal adiposity and a pro-inflammatory state in the adipose tissue (and in general). In murine and human studies investigating supplementation of VLCM *n*-3 PUFAs, a tendency to change from a pro-inflammatory state into an anti-inflammatory state of the adipose tissue has been shown [43,44]. Thus, a transition of macrophages from the pro-inflammatory M1-type to the anti- inflammatory M2-type in response to VLCM *n*-3 PUFAs has been described [45]. In vitro cellular studies have demonstrated that these metabolic effects are partly mediated by peroxisome–proliferator–activator receptor (PPAR) transcription factors, typically with two receptor subtypes, PPARα and PPARγ. Whereas PPARα is also thought to mediate the beneficial effect of VLCM *n*-3 PUFAs on hepatic insulin sensitivity, PPARγ mediates the effect on adiponectin upregulation [44]. Both subtypes are also known to counteract the activation of the key proinflammatory transcription factor NFkB [12,13,15,44,46] (Figure 2).

Another rapidly evolving area has been the role of micro-RNAs (miRNAs) in the inflammatory balance in white adipose tissue, and their possible anti-inflammatory role, also related to the presence of insulin resistance [43]. Protective effects of VLCM *n*-3 PUFAs has also been linked to certain miRNAs, and the importance of the ratio between *n*-6 and *n*-3 FAs in the diet, as well as in circulation and adipose tissue, has been emphasized [43].

**Figure 3:**
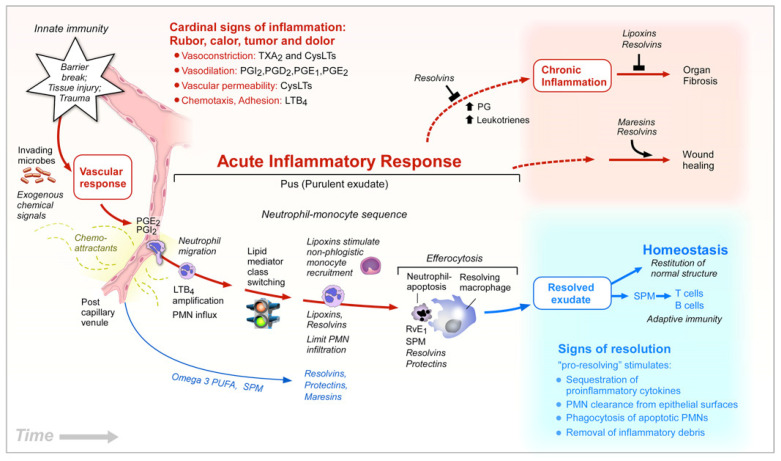
Lipid mediators in the acute inflammatory response, resolution, and other outcomes. Copied from Serhan CN, *Nature* **2014**, *510*, 92–101 [18], with permission.

Regarding adiposity per se, especially abdominal adiposity, the influence of VLCM *n*-3 PUFAs and the *n*-6/*n*-3 ratio has mainly been focused on the pro-inflammatory state and insulin resistance. It emerges from both animal and human studies that the modern Western diet with increased *n*-6/*n*-3 ratio is associated with increased incidence of adiposity, and increased pro-inflammatory activity in the adipose tissue, increased insulin resistance, and increased risk of type 2 diabetes. Results from studies with supplementation of VLCM *n*-3 PUFAs in the diet or as purified supplements indicate that reducing the *n*-6/*n*-3 ratio counteracts adiposity and normalizes the dysmetabolic state in the metabolic syndrome [43,46,47,48,49]. This effect may be linked to the anti-inflammatory properties of VLCM *n*-3 PUFAs by their role in resolution of inflammation (Figure 3).

Additionally, the possible importance of polymorphisms in the regulation of fatty acids desaturases (FADS) for the *n*-6/*n*-3 ratio has been focused on [50]. Specific clustering of these polymorphisms on chromosome 11 seems further to be related to sex and compliance with a Mediterranean diet. 

## 3. VLCM *n*-3 PUFAs Role in Cardiovascular Risk Assessement

Since the Diet and Reinfarction Trial (DART) in 1989 found a 29% reduction in 2-year mortality associated with increased fish intake (fatty fish twice per week) in approximately 2000 post-MI patients [51], several randomized clinical trials have investigated the effect of VLCM *n*-3 PUFA in reducing cardiovascular risk. Most studies have been performed with use of supplements. The GISSI-Prevenzione trial, also a secondary prophylaxis study, demonstrated in 1999 a significant reduction in all-cause mortality (21%) and 45% reduction in sudden cardiac death in ~11,000 patients randomized to 0.85 g EPA + DHA/day versus placebo [33]. The first large RCT testing EPA alone was the Japan EPA Lipid Intervention Study (JELIS), randomizing more than 18,000 individuals already treated with statins, to 1.8 g EPA/day versus placebo for 5 years [52]. They found a 19% reduction in major coronary events, but no significant reduction in CV death. Interestingly, patients with hypertriglyceridemia were identified as super-responders, with a 53% reduction in major coronary events. In the last decade, several studies have investigated low-dose EPA + DHA in secondary prevention without any convincing cardiovascular benefit [53,54,55,56].

In 2018, three much anticipated RCTs on primary prevention were published. The Vitamin D and Omega-3 Trial (VITAL) randomized almost 26,000 healthy participants to 1 g EPA + DHA/day and followed them for 5 years [57]. They did not find any significant reduction in the composite endpoint of AMI, stroke, and CV death. The ASCEND (A Cardiovascular Study of Events in Diabetes) Study included more than 15,000 patients with diabetes mellitus and randomized them to ~1 g of EPA + DHA/day versus placebo [58]. Also in this study, there was no significant reduction in cardiovascular events over 7 years of follow-up. The third study was markedly different in design and treatment. The Reduction of Cardiovascular Events with Icosapent Ethyl-Intervention Trial (REDUCE-IT) tested 4 g/day of icosapent ethyl versus placebo in 8179 statin-treated adults with hypertriglyceridemia [37]. A striking 25% reduction in the primary composite endpoint of CV death, nonfatal MI, stroke, coronary revascularization, and unstable angina was observed after 5 years. 

A separate mechanistic RCT (EVAPORATE) used serial coronary CT scans to evaluate changes in coronary plaque burden in REDUCE-IT-like patients with 4 g/day of icosapent ethyl [59]. This study showed significant reduction in the primary endpoint of changes in low-attenuating plaque volume (−17% vs. +109% in the placebo group), and a significant reduction also in fibro-fatty plaques (−34% vs. +32%) but not in calcified plaques (−1% vs. +15%) after 18 months. Based on these studies, and category A science Advisory from the American Heart Association [40], the Food and Drug Administration (FDA) in 2019 approved icosapent ethyl as an adjunctive therapy to reduce the risk of CV events in patients with hypertriglyceridemia and high risk of CVD.

During the last 30 years, we have studied a variety of effects of the VLCM *n*-3 PUFAs in randomized clinical trials as well as by use of extensive biobanks. Back in the early 1990s, the SHOT study [35] was performed to study whether supplementation with VLCM *n*-3 PUFAs (3.4 g/day of highly concentrated EPA + DHA) would positively influence the occlusion rate of coronary artery bypass grafts (CABG) compared to controls (*n* = 610). Vein graft occlusion rates after one year, assessed by angiography, were significantly lower (27%) in the VLCM *n*-3 PUFA group compared to controls (33%). Moreover, a significant trend to lower vein graft occlusion rate with increasing relative change in serum *n*-3 PUFA levels during the study period was observed. Simultaneously, serum levels of EPA increased from 38 to 92 (mg/L), and serum levels of DHA from 113 to 129 (mg/L), in the active treatment group. To the best of our knowledge, similar randomized trials have not been conducted.

In the CART study [34], performed later in the 1990s, supplementation with VLCM *n*-3 PUFAs was given daily for six months with the hypothesis to reduce the frequency of restenosis after percutaneous transluminal coronary angioplasty (PTCA). Patients were randomized to either 5.1 g/day of highly concentrated EPA + DHA or corn oil as placebo, starting two weeks prior to PTCA to allow for the FAs to be incorporated in the cell membranes before the procedure. Despite that, serum levels of EPA and DHA increased from 43 to 101 mg/L and 112 to 132 mg/L, respectively, in the VLCM *n*-3 PUFA group, no effects on the restenosis frequency were obtained (40.6% in the active treatment group vs. 35.4% in the placebo group). This is line with results from other PTCA studies conducted in the same time period using this very high dose of supplementation [60,61]. Sub-studies, however, revealed that whereas this high dose of VLCM *n*-3 PUFAs was followed by reduction in pro-thrombotic markers, markers of inflammation were increased, possibly related to lipid peroxidation visualized by reduction in vitamin E and increase in thiobarbituric acid-reactive substances [62].

The possible pro-oxidative properties of high doses of VLCM *n*-3 PUFAs have been discussed with conflicting conclusions [24,25,63,64]. This topic has mainly been explored in experimental studies, and limited in the context of patients with cardiovascular disease. In the OVITES study, 6 weeks’ worth of supplementation with either low (0.85 g/day) or high (5.1 g/day) dose of VLCM *n*-3 PUFA, or the latter amount + vitamin E 400 mg/day, was explored in high risk individuals. In the low FA group, no changes in inflammatory or peroxidation markers were observed, whereas in the high FA group alone significantly increased inflammation and peroxidation were noted, without any change when vitamin E was added [65]. Thus, the high dose of VLCM *n*-3 PUFA may induce lipid peroxidation in individuals at risk of atherosclerosis with a subsequent proinflammatory state, not counteracted by vitamin E as an antioxidant, probably because peroxidation mainly occurs at the cellular level. Nevertheless, whether higher doses have a better longstanding effect is still unclear, and is probably dependent on the population. It should be noted that daily dietary amounts in Greenland Eskimos were described as being about 10 g and even more [66]; however, this population at that time is not comparable with the general Western world population today.

In the DOIT study [36,67], the possible modulating effect of 3-year diet counselling was compared to that of supplementation with VLCM *n*-3 PUFAs given as capsules, on the progression of atherosclerosis assessed as structural measures of carotid IMT (cIMT), plaques score, and vascular function, as well as on clinical end-points. Such comparisons in clinical studies on atherosclerosis had hitherto not been extensively studied. Men at high risk for CVD were randomized in a factorial design to dietary counselling and/or VLCM *n*-3 PUFA capsules (2.4 g VLCM *n*-3 FA/day) in a placebo-controlled fashion. The dietary advices were to increase the use of vegetable oils, vegetables, fruits, and fish, and to decrease the use of meat and fat from animal sources. Reduced progression in cIMT was observed in groups with dietary counselling, whereas vascular function was significantly improved in the VLCM *n*-3 PUFA groups and significantly correlated to changes in serum EPA concentrations. When applying the so-called Plasma Index (PI) (EPA+DHA/total FAs) [22] to the *n*-3 PUFA groups, baseline levels of total FA were 5.5 g/L, PI 2.2% with EPA and 3.3% with DHA, and after 36 months total FA was 5.0 g/L. PI with EPA was increased to 4.5% and with DHA to 4.3%. Although the study was not geared toward clinical endpoints, a 50% reduction of all-cause mortality in patients randomized to VLCM *n*-3 PUFA supplementation, compared with placebo, was obtained [67].

**Figure 4:**
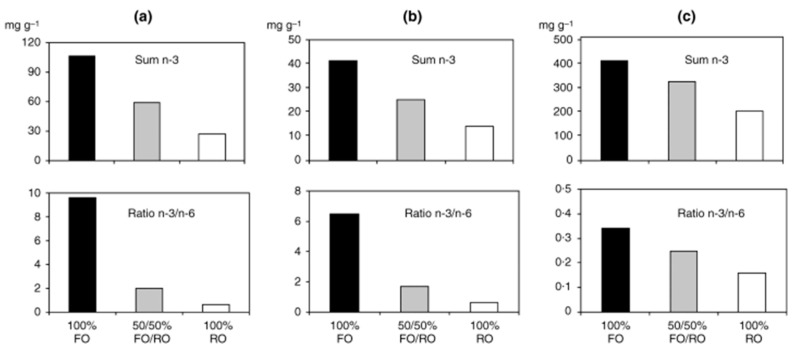
Patterns of the sum of *n*-3 FA and the ratio *n*-3/*n*-6 FA in (**a**) the three different feeds, (**b**) corresponding salmon fillets, and (**c**) serum fatty acids in patients, after the 6-week dietary intervention. FO: fish oil; RO: rapeseed oil (Seierstad SL et al. [67]).

Vegetable oils are increasingly used in fish farming industry due to shortness of marine resources; however, the effects on human health of this feeding change are largely unknown. We studied the effects of dietary intake of Atlantic salmon, fed on different fatty acid sources, on the lipid profile, vascular inflammation, and peroxidation in patients with stable coronary heart disease, in a double-blind, randomized dietary intervention study (“Fiord-to-Table”) [68,69]. Patients were randomized into three groups consuming approximately 700 g per week for 6 weeks of differently fed Atlantic salmon (fed (i) 100% South American fish oil, (ii) 50% South American fish oil/50% rapeseed oil, and (iii) 100% rapeseed oil, respectively). Significant differences between the groups in serum levels of total VLCM *n*-3 PUFA and the *n*-3/*n*-6 FA ratio were demonstrated, and interestingly, the fatty acid composition of the fish feed/pellets and fish fillets was highly mirrored in the serum FA profile of the patients after the intervention: “The patient’s serum fatty acid profile became what the salmon was fed” (Figure 4). Additionally, significant reduction in serum triglycerides and inflammatory markers were obtained in patients receiving the 100% South American fish oil.

Possible changes in dietary habits during the study period when ingestion of 700 g salmon per week was also examined [69]. In the total population, significant changes were observed for fatty fish (10.2–75.9 g/day), lean fish (18.6–0.0 g/day), processed fish (16.4–0.8 g/day), processed meat (23.1–15.8 g/day), pork (11.1–7.8 g/day), and meat stew (30.2–22.7 g/day). Thus, a healthier diet was obtained, and the beneficial results of vascular inflammation with 100% South American fish oil were conferred by the difference in feed of the farmed salmon. To the best of our knowledge, similar studies have not been conducted since.

In this context, the quality of farmed salmon on the market can be discussed, as can the toxicity of farmed fish. In this “Fiord-to-Table”study, the latter was explored, showing levels of inorganic contaminants in plasma to be only slightly changed over the study period [70].

The ongoing OMEMI Trial (NCT01841944NC) [71] is a prospective, randomized, placebo-controlled, double-blind multicenter trial, aimed to investigate the effects of supplementation with 1.8 g/day of VLCM *n*-3 PUFAs (Pikasol^®^) on cardiovascular morbidity and mortality during a follow-up period of 2 years in an elderly population (70–82 years) who have experienced an AMI. Compliance is secured by measurement of FAs in serum phospholipids. Our hypothesis is that supplementation of VLCM *n*-3 PUFAs will reduce the risk for cardiovascular events. All patients are included and follow-up is finalized. The results will be presented at the end of 2020.

## 4. Summary

The global burden of atherothrombotic heart disease should be considered a life-style disorder where differences in dietary habits and related risk factors like limited physical activity and adiposity together play important roles. Related metabolic changes have been scientifically elucidated in recent decades, and the role of VLCM FAs EPA and DHA has gained increased interest. Their possible effects on pathophysiological processes like inflammation and thrombosis have been highlighted.

In the present brief review, we have mainly focused on metabolic mechanisms in atherothrombotic heart disease, and the effects of these FAs in the clinical setting. The main focus has been on the divergent results in the field and the important differences in study population, supplements versus fresh marine sources, the proportion of EPA versus DHA, and the dosages and duration of supplementation.

We conclude that daily intake of at least 1 g of VLCM FAs may improve the dysmetabolic state. The potential to reduce the risk and progression of atherothrombotic heart disease is, however, still a matter of debate.
